# Plant-based caloric restriction diets versus conventional calorie-restricted diets for weight loss and metabolic health in obese adults: a 12-week randomized, open-label, non-inferiority trial

**DOI:** 10.3389/fnut.2026.1805225

**Published:** 2026-04-13

**Authors:** Kelibinuer Mutailipu, Liu Yang, Yaling Fang, Shihui Lei, Junwei Guo, Hang Yuan, Shuwei Liu, Yue Chen, Cuiling Zhu, Shen Qu, Falin Zhao, Le Bu

**Affiliations:** 1 Departments of Obesity and Metabolic Syndrome, Endocrinology & Metabolism, and Obesity Diagnosis and Treatment Center, Shanghai Tenth People’s Hospital, Tongji University School of Medicine, Shanghai, China; 2 Obesity Research Institute, Tongji University School of Medicine, Shanghai, China; 3 Department of Endocrinology, Sun Yat-sen Memorial Hospital, Sun Yat-sen University, Guangzhou, China; 4 School of Public Administration, Hangzhou Normal University, Hangzhou, Zhejiang, China; 5 Engineering Research Center of Mobile Health Management System, Ministry of Education, Hangzhou Normal University, Hangzhou, Zhejiang, China

**Keywords:** calorie-restricted diet, metabolic health, obesity, plant-based diet, weight loss

## Abstract

**Background:**

Plant-based caloric restriction diet (PB-CRD), combining plant-based nutrition with intermittent caloric restriction, represent a structured dietary strategy to obesity management. This study compared the effects of PB-CRD and calorie-restricted diet (CRD) on weight loss, and metabolic health, hypothesizing that PB-CRD is non-inferior to CRD for weight loss.

**Methods:**

In this 12-week, open-label, non-inferiority trial, 80 participants with obesity (body mass index [BMI]: 28–40 kg/m^2^, age: 18–45 years) were randomized in a 1:1 ratio to either a PB-CRD (‘5 + 2’ pattern: 5 days/week of provided standardized plant-based meals and 2 days/week of self-selected meals within prescribed caloric targets) or a CRD (7 days/week of self-selected meals within prescribed caloric targets). Both groups adhered to the same daily energy intake targets of 1,600 kcal/day (men) and 1,300 kcal/day (women). The primary outcome was weight loss, while secondary outcomes included body composition, glucose and lipid metabolism, liver and kidney function, oxidative stress, and inflammation markers.

**Results:**

Of the 80 participants (mean BMI: 32.03 ± 3.46 kg/m^2^; mean age: 34.78 ± 7.12 years; 31 males), 52 (65%) completed the 12-week trial (*n* = 28 in PB-CRD and *n* = 24 in CRD group). Both groups achieved significant within-group weight loss from baseline (*p* < 0.05): 5.11 kg in the CRD and 6.56 kg in the PB-CRD group. The between-group difference was −1.44 kg (95% confidence interval [CI]: −3.27 to 0.39), which remained within the pre-specified non-inferiority margin of 0.5 kg. Compared to the CRD, the PB-CRD group demonstrated significantly greater between-group reductions in total body fat percentage (2.96 [95% CI: 1.67–4.25], *p* = 0.028), uric acid, and superoxide dismutase levels, as well as increased blood urea nitrogen (*p* < 0.05). Additionally, the PB-CRD group exhibited significant within-group improvements in visceral fat mass, HOMA-IR, total cholesterol, liver function markers, and inflammatory markers (*p* < 0.05), with no substantial differences between groups.

**Conclusion:**

Both PB-CRD and CRD were effective for weight loss, with PB-CRD demonstrating non-inferiority. Furthermore, PB-CRD offered superior benefits in reducing total body fat and improving uric acid metabolism, while improving insulin sensitivity and liver function in adults with obesity.

**Clinical trial registration:**

Identifier ChiCTR1800015156.

## Introduction

Obesity is a major global public health concern, with a significant impact on health outcomes across populations (1). It is characterized by abnormal or excessive fat accumulation, influenced by genetic and environmental factors (2), and is a major risk factor for chronic diseases such as cardiovascular disease (3), type 2 diabetes (T2D) (4), hypertension (5), and certain cancers (6). The global age-standardized prevalence of obesity increased from 8.8% in 1990 to 18.5% in 2022 for women, and from 4.8 to 14.0% for men (1). In China, a comprehensive 2023 study involving 15.8 million adults found that 34.8% were overweight and 14.1% were obese (7). The rapid increase in obesity rates necessitates urgent and effective public health interventions.

Caloric restriction diet (CRD), which typically involves a 30–50% reduction in daily caloric intake, have long been recognized as an effective strategy for managing obesity (8, 9). Beyond promoting weight loss, these diets also improve metabolic health by reducing the risks of hypertension, dyslipidemia, and insulin resistance (10). Mechanistically, CRD facilitate weight loss through the creation of a negative energy balance, where energy intake is lower than energy expenditure, while simultaneously enhancing insulin sensitivity and fat oxidation. Additionally, CRD influence appetite-regulating hormones, such as leptin and ghrelin, which modulate satiety and food intake (11, 12). Recent studies demonstrate that sustained caloric restriction in non-obese individuals can positively affect metabolic rate and cardiometabolic risk factors (13). However, emerging evidence indicates that the effectiveness of CRD may be further enhanced by optimizing dietary composition, particularly through macronutrient adjustments and the inclusion of nutrient-dense foods (14, 15).

In addition to CRD, plant-based diet (PBD) have emerged as a promising strategy for managing obesity, emphasizing nutrient-dense foods such as fruits, vegetables, whole grains, legumes, nuts, and seeds (16). Studies indicate that PBD are associated with significant weight loss, improved lipid profiles, and enhanced insulin sensitivity, which collectively reduce the risk of cardiovascular disease (17, 18). These benefits are largely attributed to the high fiber content of plant-based foods and the avoidance of energy-dense foods (19, 20). Recent studies also emphasize the importance of calorie quality over quantity, suggesting that PBD can improve metabolic fuel utilization and shift calorie partitioning away from fat storage (21). A study involving Chinese adults with prediabetes found that combining caloric restriction with PBDs resulted in comparable weight loss and improved glucose control (22). Therefore, integrating plant-based caloric restriction diet (PB-CRD) may offer a potentially complementary approach, helping to overcome the limitations of traditional CRD while providing additional metabolic benefits.

Despite the established benefits of both CRD and PBD on weight loss and metabolic health, few studies have directly compared the effects of combining these two approaches. Given that conventional CRD is a widely recognized and effective intervention, we utilized a non-inferiority design to determine whether a structured ‘5:2’ PB-CRD protocol could serve as a clinically comparable and viable alternative. We hypothesized that the PB-CRD would be non-inferior to the traditional CRD in promoting weight loss, while potentially offering supplementary metabolic benefits.

## Methods

### Study design and participants

This single-center, non-inferiority, parallel-group randomized controlled trial was conducted at the Department of Endocrinology, Shanghai Tenth People’s Hospital, between October 20, 2022, and November 20, 2023. Participants were randomly assigned in a 1:1 ratio to either the PB-CRD or CRD group for a 12-week intervention. The trial protocol is available in Supplementary material S1.

Eligible participants in the study were adults aged 18 to 45 years with a body mass index (BMI) of 28 to 40 kg/m^2^, as defined by the Chinese obesity guidelines (23). Exclusion criteria were as follows: (1) diagnoses of type 1 diabetes, T2D, gestational diabetes, or other specific diabetes subtypes; (2) a history of weight-loss surgery, use of weight-loss medications within the past 90 days, or self-reported weight changes of ≥5 kg within that period; (3) severe psychiatric disorders, such as major depression or schizophrenia; (4) uncontrolled thyroid disease, a history of malignancy in the past 5 years, or recent cardiovascular events; (5) pregnancy, breastfeeding, or plans to conceive; and (6) substance abuse or participation in other clinical trials within the past 3 months.

The study protocol was approved by the Ethics Committee of Shanghai Tenth People’s Hospital (approval number: SHYS-IEC-5.0/22 K268/P01) and registered at the China Clinical Trial Registration Center (registration number: ChiCTR1800015156). All participants provided written informed consent before enrollment.

### Randomization and blinding

Participants were randomly assigned to either the PB-CRD or CRD group in a 1:1 ratio. The random sequence generation was performed using SPSS version 23.0 by an independent researcher. For allocation concealment and implementation, this independent researcher, who was not involved in participant enrollment or clinical assessment, held the predetermined randomization list. After a participant was deemed eligible and provided informed consent, the clinical coordinator obtained the group assignment from the independent researcher. Although the trial was open-label, outcome assessors and data analysts were blinded to the group allocations to mitigate potential bias.

### Diet interventions

This study included two dietary interventions: the PB-CRD and the CRD. Both interventions adhered to a continuous daily caloric restriction protocol, targeting a 25% reduction in daily caloric intake, with energy goals of 1,300 kcal/day for women and 1,600 kcal/day for men, in accordance with the 2022 Dietary Guidelines for Chinese Residents (24).

Participants in the PB-CRD group followed a 5:2 meal-provision pattern under continuous caloric restriction. For 5 days per week, they consumed three standardized plant-based meals provided by Meat Fresh Biotechnology, comprising 55–60% carbohydrates, 25–30% fats, and 15–20% proteins (detailed composition in Supplementary material S1). Participants were required to strictly adhere to the study-provided meals without additional food intake to meet prescribed caloric targets. On the two self-selected days (weekends), participants selected their meals based on their initial nutritional training while strictly maintaining the overall caloric restriction targets. This intermittent approach aimed to mitigate potential nutrient deficiencies, particularly in calcium and omega-3 fatty acids, by allowing dietary diversity (16, 18). Conversely, participants in the CRD group selected their meals daily based on personal dietary preferences while adhering to the same caloric limits, with no specific macronutrient restrictions to ensure dietary flexibility.

Prior to the intervention, all participants received training from nutritionists on portion control and dietary management to facilitate compliance, particularly on self-selected days. Adherence was monitored daily via a mobile app, where participants uploaded real-time meal photos using their fist as a standardized size reference—a pragmatic, intra-individually consistent visual scale widely adopted in public health guidelines to facilitate compliance. To mitigate recall bias, nutritionists provided weekly feedback based on real-time caloric evaluations. Participants with a meal-logging rate below 50% were discontinued. No specific exercise guidelines were issued, with participants maintaining usual activity levels.

### Outcomes and follow-up

The primary outcome was the change in body weight from baseline to the end of the 12-week intervention. Secondary outcomes included changes in body composition, metabolic risk factors, liver and renal function parameters, antioxidant levels, and inflammatory markers.

Body composition was assessed using the InBody analyzer and iDXA system (Lunar, GE Healthcare). Measured parameters included total body fat percentage (TBF%), visceral fat area (VFA), estimated visceral fat mass (EVFM), estimated visceral fat volume (EVFV), estimated VFA (EVFA), skeletal muscle mass, body fat mass, fat mass index, android/gynoid ratio, trunk/limb ratio, trunk/quad ratio, lean BMI (LBMI), and fat-free mass index. Metabolic risk factors were assessed using an oral glucose tolerance test, measuring blood glucose (BG), insulin, and C-peptide levels at fasting, 0.5, 1, 2, and 3 h, along with serum lipids (total cholesterol [TC], triglycerides, low-density lipoprotein, high-density lipoprotein). Area under the curve (AUC) values were calculated for blood glucose (AUCBG), insulin (AUCins), and C-peptide (AUCC), as well as the AUCins/AUCBG ratio. In addition, indices for insulin resistance and insulin sensitivity were calculated, including Homeostasis Model Assessment of Insulin Resistance (HOMA-IR), Homeostasis Model Assessment of Beta Cell Function (HOMA-*β*), and Matsuda Index. The formulas used for calculating these indices are provided in Supplementary methods S1. Kidney function was evaluated by uric acid (UA) and blood urea nitrogen (BUN), while liver function was assessed by alanine aminotransferase (ALT), aspartate aminotransferase (AST), gamma-glutamyl transferase (GGT), and alkaline phosphatase (ALP). Antioxidant and inflammatory markers included superoxide dismutase (SOD), interleukin-6 (IL-6), IL-8, and tumor necrosis factor-*α* (TNF-α). All outcomes were measured at baseline and after the 12-week intervention.

### Safety and adverse event monitoring

Adverse events (AEs) were monitored throughout the study via weekly follow-up interviews and participant self-reporting. AEs were defined as any adverse medical events during the study, and their severity was assessed according to the Common Terminology Criteria for Adverse Events (CTCAE) version 5.0. The monitoring focused on potential diet-related side effects, including gastrointestinal symptoms (e.g., abdominal pain, dyspepsia, or constipation) and general systemic reactions (e.g., fatigue, dizziness, or headache).

### Statistical analysis

Sample size estimation was conducted using PASS software, based on standard deviations obtained from a preliminary trial: 2.5 kg for the PB-CRD group and 4 kg for the CRD group. Based on a pilot study, we anticipated a true mean difference of 2.0 kg in weight reduction. The non-inferiority margin was set at 0.5 kg, a difference considered clinically negligible in 12-week weight-loss interventions and equivalent to one-fifth of the PB-CRD group’s standard deviation. Using a two-sided significance level (*α*) of 0.05 and a power of 90% and after accounting for a 20% dropout rate, the final sample size was determined to be 40 participants per group.

Data were managed using Epidata 3.1, with statistical analyses performed in SPSS version 20.0 (SPSS Inc., Chicago, IL, USA), and graphical representations created using GraphPad Prism version 9.1. Continuous variables were tested for normality using the Shapiro–Wilk test, and those following a normal distribution (e.g., body weight, BMI) are expressed as means and standard deviations, with group comparisons using independent t-tests. Non-normally distributed variables (e.g., IL-8 and fasting insulin) are described as medians and interquartile ranges and analyzed using the Mann–Whitney U test. Categorical variables are reported as counts and percentages, with group comparisons performed using chi-square tests.

Within-group changes from baseline to the intervention endpoint are presented as means with 95% confidence intervals (CIs). Paired t-tests were used for normally distributed differences, while the Wilcoxon signed-rank test was applied for non-normally distributed differences. A two-tailed significance level of 0.05 was applied for all analyses. Non-inferiority was assessed using a predefined margin (*Δ* = 0.5Kg). By treating weight reduction as a positive value, non-inferiority was established if the upper bound of the two-sided 95% CI for the mean difference (CRD minus PB-CRD) was less than 0.5 kg (△).

For the primary outcome, analyses were performed using both the intention-to-treat (ITT) and the per-protocol set (PPS) approaches. In the ITT analysis, missing values were handled using the Last Observation Carried Forward (LOCF) method. The ITT analysis encompassed all randomized participants, while the PPS analysis included only those who adhered to the study protocol and completed the intervention. Secondary outcomes were assessed exclusively at baseline and the study endpoint. As data from participants who did not complete the study were unavailable, only PPS analysis results were reported for these outcomes.

## Results

### Baseline characteristics

**Figure 1:**
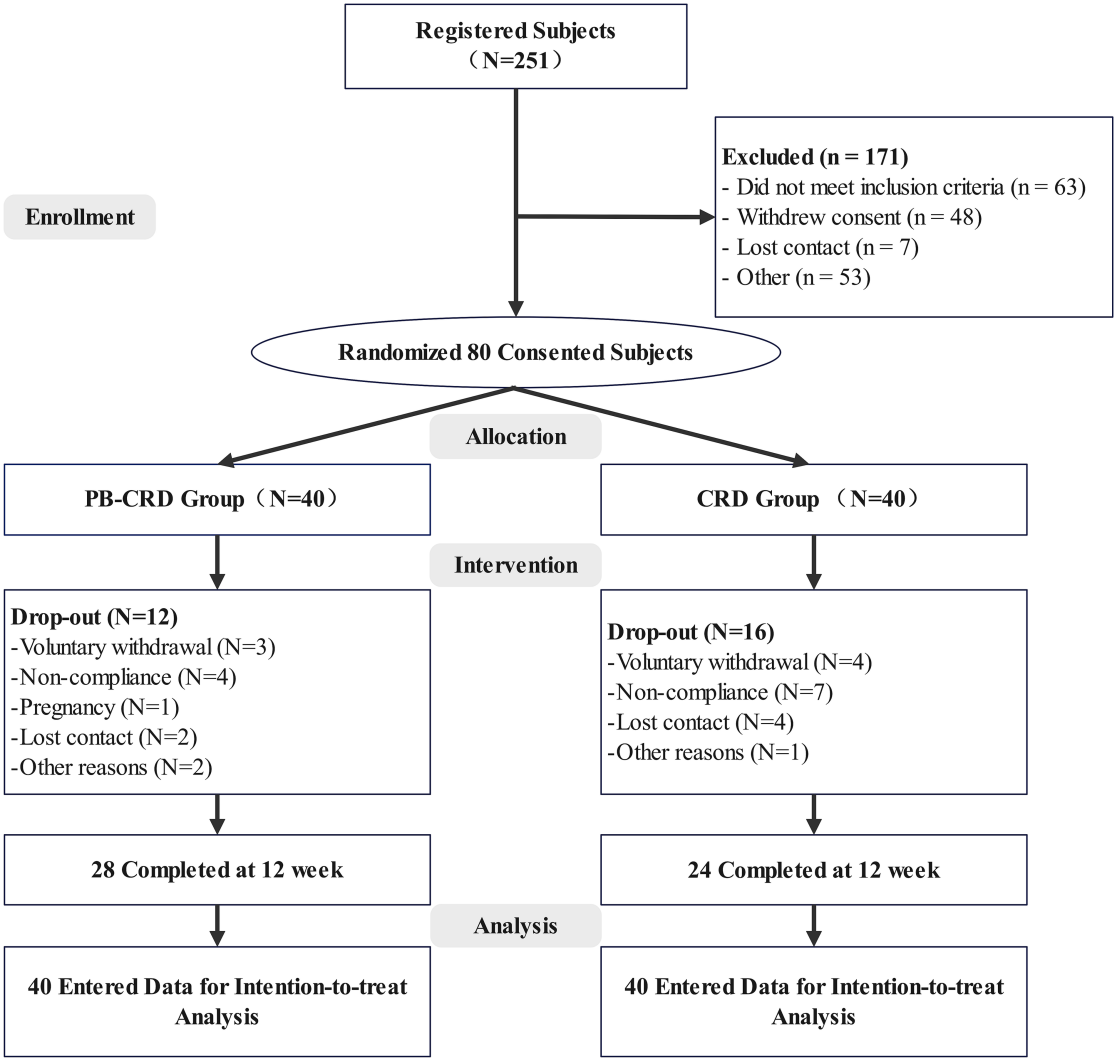
CONSORT diagram of participant flow. PB-CRD, plant-based calorie-restricted diet; CR, calorie-restricted diet.

A total of 251 patients were screened, and 80 were randomized to either the PB-CRD group (*n* = 40) or the CRD group (*n* = 40). Among those randomized, 28 participants in the PB-CRD group and 24 in the CRD group completed the 12-week intervention, resulting in a total attrition rate of 35% (Figure 1). The mean age of participants was 34.78 ± 7.12 years, with a mean BMI of 32.03 ± 3.46 kg/m^2^. Males comprised 38.8% of the total participants (Table 1). All baseline clinical and biochemical parameters were well-balanced between the two groups (all *p* > 0.05). Dropout rates were comparable between the groups, with no statistically significant difference observed, and details for dropout are provided in Supplementary Tables S1, S2.

**Table 1:** Baseline characteristics of study participants.

Characteristic	Total (*N* = 80)	PB-CRD (*N* = 40)	CRD (*N* = 40)
Sex (Male/%)	31/38.8	19/47.5	12/30.0
Age (Years)	34.78 ± 7.12	35.38 ± 8.01	34.17 ± 6.14
BMI (kg/m2)	32.03 ± 3.46	31.91 ± 3.38	32.15 ± 3.58
WC (cm)	100.06 ± 11.64	100.31 ± 10.99	99.81 ± 12.40
WHR	0.91 ± 0.08	0.92 ± 0.07	0.90 ± 0.08
SBP (mmHg)	135.08 ± 15.21	134.50 ± 13.96	135.68 ± 16.58
DBP (mmHg)	88.32 ± 10.70	87.63 ± 11.01	89.03 ± 10.48
HR (Beats/Minute)	85.00 ± 6.24	83.61 ± 7.45	86.47 ± 4.25
SF-36 Physical Health	48.87 ± 4.98	49.41 ± 4.09	48.30 ± 5.77
SF-36 Mental Health	47.74 ± 6.62	48.26 ± 6.56	47.19 ± 6.71
IWQOL-lite CT Score	123.87 ± 22.20	128.08 ± 16.11	119.55 ± 26.60
Sleep Disturbance Score	8.25 ± 3.84	8.31 ± 3.25	8.19 ± 4.41
PHQ-9	5.76 ± 4.69	4.64 ± 3.04	6.89 ± 5.75
BECK Depression Inventory	4 (2–7)	4 (1.25–5.75)	6 (2–8.75)
BFM (Kg)	35.95 ± 0.84	35.65 ± 7.75	36.24 ± 8.42
VFA (cm^2^)	168.42 ± 38.37	165.09 ± 38.34	171.56 ± 38.76
SMM (Kg)	31.16 ± 7.36	32.38 ± 7.32	30.01 ± 7.32
Inbody Score	63.78 ± 8.46	64.83 ± 6.33	62.81 ± 10.07
TBF (%)	42.46 ± 5.29	41.49 ± 4.88	43.48 ± 5.57
FMI (kg/m^2^)	13.31 ± 2.28	13.15 ± 2.28	13.47 ± 2.29
A/G Ratio	1.16 ± 0.17	1.18 ± 0.17	1.15 ± 0.18
T/L Ratio	1.11 ± 0.15	1.12 ± 0.14	1.09 ± 0.15
T/Q Ratio	1.21 ± 0.25	1.26 ± 0.26	1.15 ± 0.22
LBMI (kg/m^2^)	16.87 ± 2.28	17.12 ± 2.31	16.63 ± 2.24
EVFM (g)	860.03 ± 204.57	857.32 ± 196.13	862.74 ± 215.28
EVFV (cm^3^)	930.22 ± 221.67	927.74 ± 213.09	932.71 ± 232.77
EVFA (cm^2^)	178.43 ± 42.51	181.08 ± 45.13	175.65 ± 39.99
FFMI (Kg/m^2^)	7.77 ± 3.57	8.1 ± 4.77	7.4 ± 1.65
FT3 (pmol/L)	5.49 ± 0.62	5.59 ± 0.54	5.39 ± 0.68
FT4 (pmol/L)	16.01 ± 1.97	16.35 ± 1.68	15.65 ± 2.21
TSH (mU/L)	2.74 ± 1.25	2.59 ± 1.09	2.91 ± 1.40
hbA1c (%)	5.51 ± 0.37	5.48 ± 0.36	5.5 ± 0.37
FBG (mmol/l)	4.92 ± 0.48	4.95 ± 0.53	4.88 ± 0.430
0.5hBG (mmol/l)	8.87 ± 1.594	8.95 ± 1.65	8.80 ± 1.56
1hBG (mmol/l)	8.84 ± 2.34	8.73 ± 2.20	8.93 ± 2.51
2hBG (mmol/l)	6.89 ± 1.62	6.85 ± 1.34	6.94 ± 1.87
3hBG (mmol/l)	4.67 ± 1.03	4.71 ± 1.10	4.64 ± 0.98
FINS (mU/L)	17.83 (13.28–23.79)	17.53 (13.28–24.54)	18.11 (13.15–22.89)
0.5hINS (mU/L)	124.08 ± 66.89	125.01 ± 70.16	123.16 ± 64.36
1hINS (mU/L)	127.60 (68.84–194.45)	128.40 (65.23–217.20)	126.80 (74.37–189.90)
2hINS (mU/L)	76.28 (49.83–151.10)	73.17 (49.38–148.50)	81.01 (57.07–151.50)
3hINS (mU/L)	17.45 (9.96–37.67)	17.77 (8.80–33.19)	17.32 (10.40–38.94)
FCP (ng/mL)	3.07 (2.53–3.92)	3.09 (2.53–4.05)	3.03 (2.53–3.82)
0.5hCP (ng/mL)	9.24 (7.25–12.00)	8.99 (7.26–12.09)	9.45 (7.13–11.97)
1hCP (ng/mL)	11.61 ± 4.09	11.97 ± 4.53	11.25 ± 3.64
2hCP (ng/mL)	10.57 ± 4.01	10.39 ± 4.00	10.76 ± 4.73
3hCP (ng/mL)	4.88 (3.53–6.46)	4.97 (3.53–6.45)	4.58 (3.44–7.25)
UA (umol/L)	412.98 ± 109.47	427.66 ± 115.27	395.72 ± 101.18
BUN (mmol/l)	4.25 ± 1.04	4.35 ± 1.16	4.14 ± 0.89
TC (mmol/L)	5.01 ± 0.92	5.04 ± 0.86	4.98 ± 1.00
TG (mmol/L)	1.65 ± 1.01	1.59 ± 0.89	1.72 ± 1.15
HDL (mmol/L)	1.09 ± 0.22	1.08 ± 0.23	1.10 ± 0.22
LDL (mmol/L)	3.17 ± 0.80	3.24 ± 0.74	3.10 ± 0.88
ALT (U/L)	31.70 (20.20–55.35)	36.70 (20.93–67.05)	26.40 (19.30–52.25)
AST (U/L)	21.00 (16.80–31.80)	21.65 (18.43–34.75)	19.50 (16.20–30.05)
GGT (U/L)	30.65 (20.65–56.18)	28.00 (21.40–39.90)	31.60 (19.70–66.70)
ALP (U/L)	67.23 ± 18.46	66.25 ± 21.07	68.35 ± 15.15
SOD (U/mL)	246.94 ± 45.76	253.67 ± 21.37	240.21 ± 60.95
IL-6	2.40 (2.00–3.62)	2.00 (2.00–3.52)	2.00 (2.00–3.47)
IL-8	65.17 ± 82.50	77.78 ± 101.96	52.56 ± 55.65
TNF	20.30 (11.25–37.95)	19.30 (9.03–42.50)	22.35 (14.85–30.83)

Data are express as means ± SD (standard deviation), or number (%). *p*-values refer to t-tests for continuous variables and χ2 (chi-squared) test for categorical variables. BMI, body mass index; WC, waist circumference; WHR, waist-to-hip ratio; SBP, systolic blood pressure; DBP, diastolic blood pressure; HR, heart rate; VFA, visceral fat area; SMM, skeletal muscle mass; TBF, total body fat percentage; FMI, fat mass index; A/G Ratio, android to gynoid fat ratio; T/L Ratio, trunk to lower extremity fat ratio; T/Q Ratio, trunk to quadrants fat ratio; LBMI, lean body mass index; EVFM, estimated visceral fat mass; EVFV, estimated visceral fat volume; EVFA, estimated visceral fat area; FFMI, fat-free mass index; FT3, free triiodothyronine; FT4, free thyroxine; TSH, thyroid-stimulating hormone; hbA1c, hemoglobin A1c; FBG, fasting blood glucose; 0.5hBG, 0.5-h blood glucose; hBG, 1-h blood glucose; 2hBG, 2-h blood glucose; 3hBG, 3-h blood glucose; FINS, fasting insulin; 0.5hINS, 0.5-h insulin; 1hINS, 1-h insulin; 2hINS, 2-h insulin; 3hINS, 3-h insulin; FCP, fasting C-peptide; 0.5hCP, 0.5-h C-peptide; 1hCP, 1-h C-peptide; 2hCP, 2-h C-peptide; 3hCP, 3-h C-peptide; TC, total cholesterol; TG, triglycerides; HDL, high-density lipoprotein cholesterol; LDL, low-density lipoprotein cholesterol; UA, uric acid; BUN, blood urea nitrogen; ALT, alanine aminotransferase; AST, aspartate aminotransferase; GGT, gamma-glutamyl transferase; ALP, alkaline phosphatase; SOD, superoxide dismutase; IL-6, interleukin-6; IL-8, interleukin-8; TNF, tumor necrosis factor.

### Weight loss

**Figure 2:**
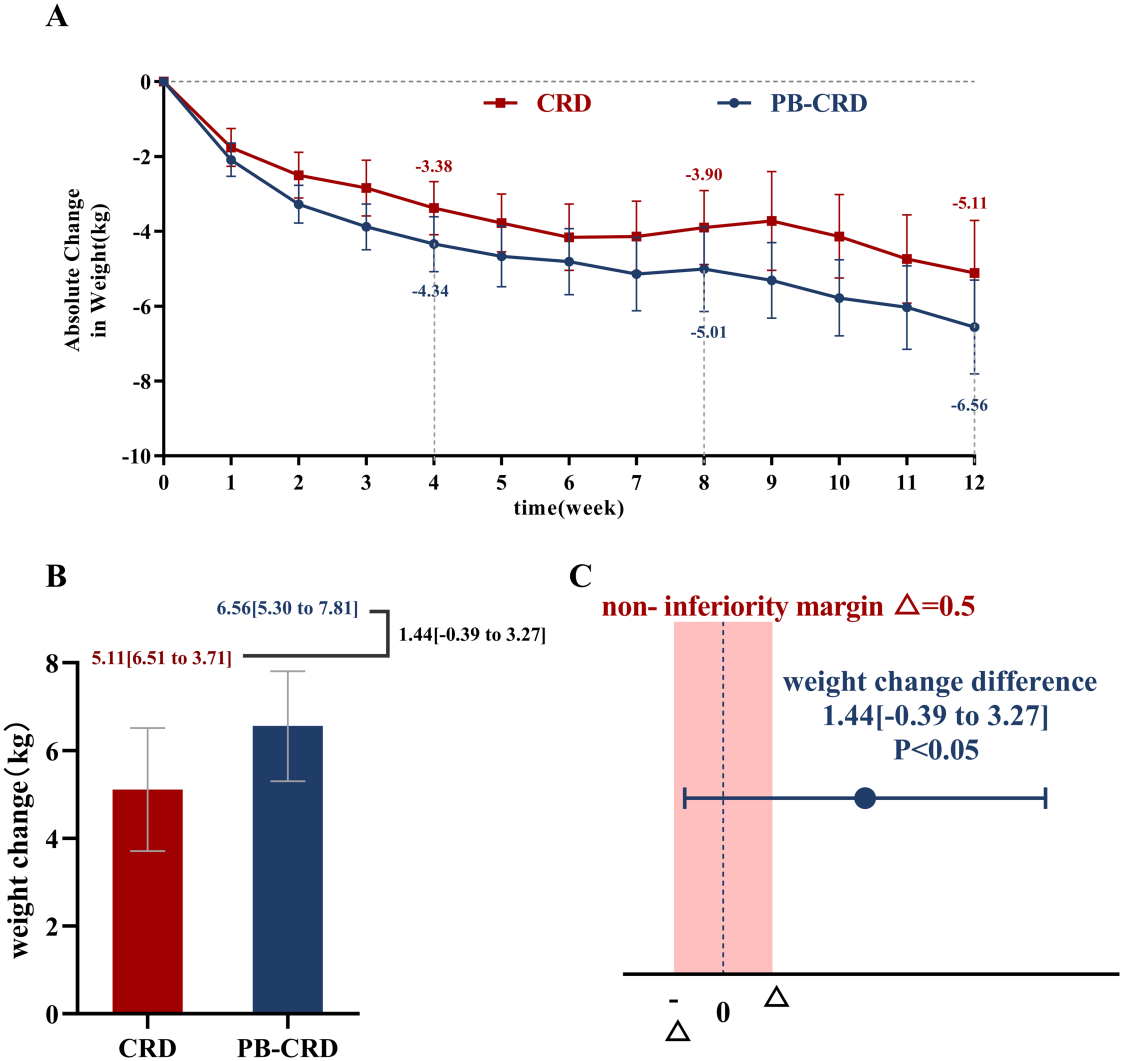
Effects of PB-CRD and CRD on body weight. **(A)** Absolute weight change over time; **(B)** total weight change after 12 weeks; **(C)** non-inferiority analysis of weight change difference.

In the PPS analysis (Figure 2), both the PB-CRD and CRD groups demonstrated significant weight reductions over 12 weeks. The mean weight loss was 5.11 kg (95% CI: 3.71 to 6.51) in the CRD group and 6.56 kg (95% CI: 5.30 to 7.81) in the PB-CRD group. The between-group difference was −1.44 kg (95% CI: −3.27 to 0.39), which fell within the pre-specified non-inferiority margin of 0.5 kg. Non-inferiority testing confirmed that the PB-CRD group was not inferior to the CRD group in terms of weight loss (*p* = 0.019). Results from the ITT analysis (Supplementary Figure S1) showed a reduced difference between the two groups, with a mean weight change difference of −0.82 kg (95% CI: −2.16 to 0.51; *p* = 0.317), representing an attenuated effect size compared to the PPS analysis due to the dilution effect of dropouts. Supplementary analyses provide further characterization of the dropout population, their weight changes, and the subsequent impact on the primary endpoint (Supplementary Figure S2; Supplementary Tables S3–S5).

### Body composition

Table 2 shows that after 12 weeks, the PB-CRD group exhibited significantly greater improvements in body composition compared to the CRD group. Specifically, the PB-CRD group had a more substantial reduction in TBF% (2.96, 95% CI: 1.67 to 4.25) compared to the CRD group (0.81, 95% CI: −0.68 to 2.30), with a significant between-group difference (*p* = 0.028). Furthermore, the PB-CRD group demonstrated significant reductions in EVFM (122.80 g, 95% CI: 53.91 to 191.69), EVFV (132.80 cm^3^, 95% CI: 58.44 to 207.16), and EVFA (25.40 cm^2^, 95% CI: 11.14 to 39.66). In contrast, no significant changes were observed in the CRD group for these parameters, and no between-group differences were found.

**Table 2:** Changes in body composition parameters from baseline to week 12.

Variable	PB-CRD (*N* = 24)			CRD group (*N* = 28)			*p* value^b^
	Baseline	12-week	Change (Baseline - Week 12)	Baseline	12-week	Change (Baseline - Week 12)	
BFM (Kg)	32.32 ± 6.08	28.86 ± 7.12	3.46 (1.58–5.34)^a^	36.75 ± 8.57	32.93 ± 8.65	3.83 (2.48–5.17)^a^	0.741
VFA (cm^2^)	151.49 ± 35.04	133.57 ± 38.80	17.92 (8.75–27.09)^a^	173.51 ± 40.62	156.54 ± 42.85	16.98 (9.82–24.13)^a^	0.866
SMM (Kg)	30.94 ± 1.48	30.48 ± 1.33	0.46 (−0.12–1.04)	30.86 ± 7.34	30.03 ± 6.86	0.83 (−0.53–2.19)	0.587
TBF (%)	40.79 ± 5.06	37.82 ± 5.61	2.96 (1.67–4.25)^a^	43.09 ± 6.30	42.28 ± 6.83	0.81 (−0.68–2.30)	0.028*
FMI (kg/m^2^)	12.66 ± 2.24	11.15 ± 2.48	1.51 (0.97–2.05)^a^	13.71 ± 2.62	12.66 ± 2.50	1.05 (0.51–1.59)^a^	0.217
A/G Ratio	1.19 ± 0.17	1.16 ± 0.15	0.02 (−0.02–0.07)	1.15 ± 0.17	1.09 ± 0.13	0.05 (0.01–0.10)^a^	0.324
T/L Ratio	1.13 ± 0.13	1.08 ± 0.15	0.04 (0.01–0.07)^a^	1.09 ± 0.17	1.05 ± 0.13	0.04 (0.01–0.06)^a^	0.884
T/Q Ratio	1.22 ± 0.18	1.17 ± 0.18	0.05 (−0.02–0.12)	1.13 ± 0.18	1.12 ± 0.19	0.01 (−0.04–0.05)	0.296
LBMI (kg/m^2^)	17.23 ± 1.93	17.09 ± 1.79	0.14 (−2.08–0.52)	17.00 ± 2.33	16.12 ± 2.34	0.89 (0.14–1.63)^a^	0.060
EVFM (g)	854.45 ± 145.83	731.65 ± 145.36	122.80 (53.91–191.69)^a^	882.06 ± 169.27	805.22 ± 240.87	76.83 (−11.06–164.73)	0.388
EVFV (cm^3^)	923.75 ± 157.10	790.95 ± 157.10	132.80 (58.44–207.16)^a^	953.61 ± 183.17	870.50 ± 260.62	83.11 (−11.99–178.22)	0.388
EVFA (cm^2^)	177.20 ± 30.26	151.81 ± 30.13	25.40 (11.14–39.66)^a^	182.89 ± 35.14	167.11 ± 50.02	15.78 (−2.43–33.98)	0.383
FFMI (Kg/m^2^)	7.56 ± 0.94	7.43 ± 0.93	0.13 (−0.14–0.40)	7.84 ± 1.93	7.00 ± 1.46	0.83 (−0.09–1.77)	0.841

VFA, visceral fat area; SMM, skeletal muscle mass; TBF, total body fat percentage; FMI, fat mass index; A/G Ratio, android to gynoid fat ratio; T/L Ratio, trunk to lower extremity fat ratio; T/Q Ratio, trunk to quadrants fat ratio; LBMI, lean body mass index; EVFM, estimated visceral fat mass; EVFV, estimated visceral fat volume; EVFA, estimated visceral fat area; FFMI, fat-free mass index.

Changes from baseline to week 12 expressed with 95% confidence intervals (CI) *p*-values evaluate the statistical significance of differences between groups.

^a^*p* < 0.05 for within-group changes from baseline, assessed by paired *t*-tests.

^b^*p* values are for the interaction between group, assessed by repeated measures analysis of variance (ANOVA), *p < 0.05.

### Glucose and lipid metabolism

The PB-CRD group showed significant improvements in glucose metabolism. Specifically, BG levels were significantly reduced at 30 min, and the AUCBG was significantly lower (2.06 [95% CI: 0.25 to 3.87], *p* = 0.027). Insulin levels did not show significant changes, although AUCins approached statistical significance (*p* = 0.052). C-peptide levels decreased significantly at all time points (*p* < 0.05), and AUCC-peptide was significantly reduced (13.89 [95% CI: 5.45 to 22.32], *p* = 0.002). Additionally, insulin sensitivity improved significantly, as evidenced by a decrease in HOMA-IR (*p* = 0.011), an increase in the Matsuda index (*p* = 0.025), and a reduction in AUCins/AUCBG (*p* = 0.035). However, insulin secretion capacity, measured by HOMA-*β*, did not show significant changes (*p* = 0.303). Regarding lipid metabolism, the PB-CRD group showed a significant reduction in TC (0.25 [95% CI: 0.01 to 0.48], *p* = 0.035).

**Figure 3:**
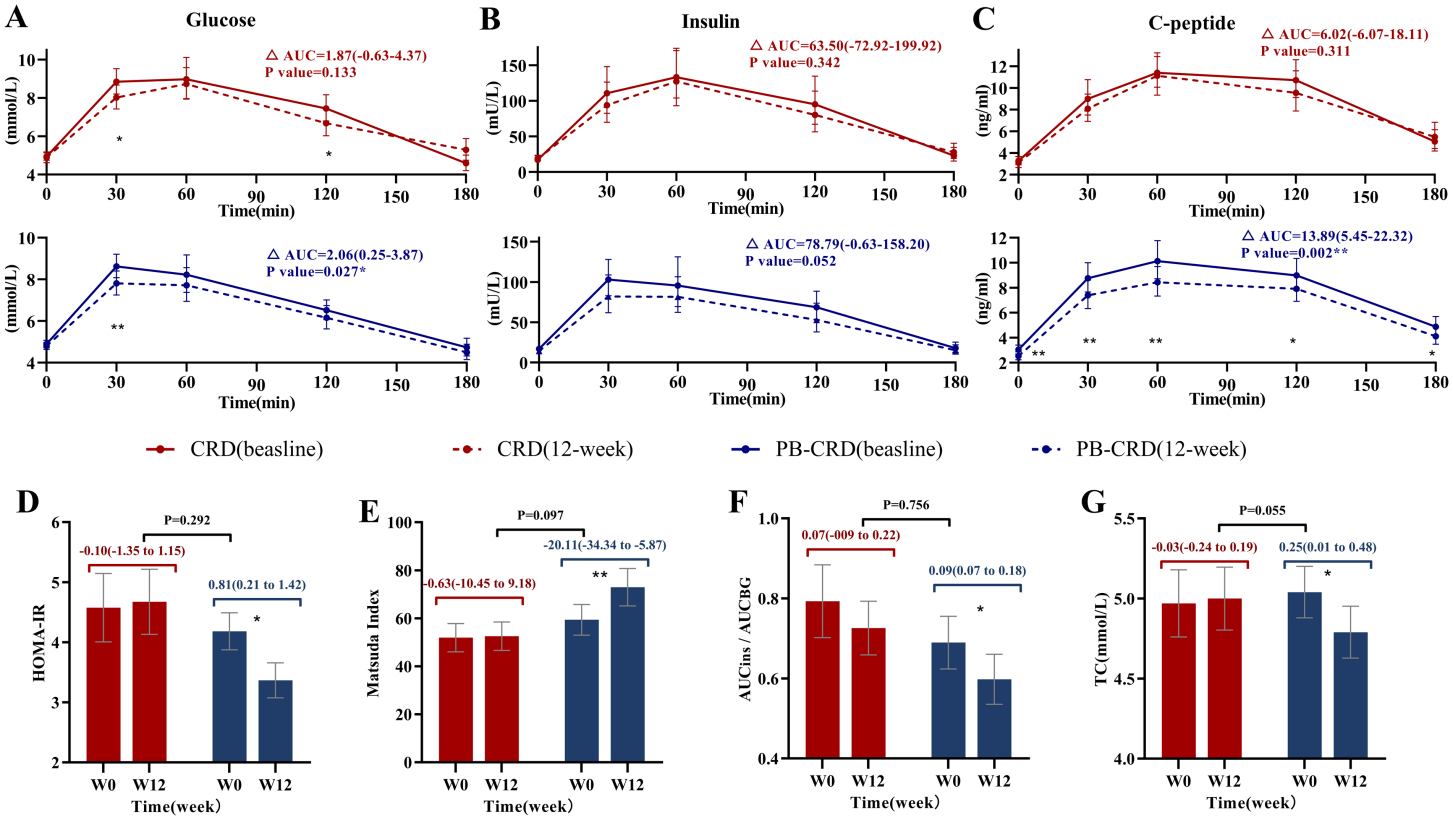
Changes in metabolic parameters from baseline to week 12. **(A)** Area under the curve for blood glucose (AUCBG); **(B)** area under the curve for insulin (AUCins); **(C)** area under the curve for C-peptide (AUCC); **(D)** Homeostasis Model Assessment of Insulin Resistance (HOMA-IR); **(E)** Matsuda index; **(F)** ratio of AUCinsulin to AUCglucose (AUCins/AUCBG); **(G)** total cholesterol (TC); **p < 0.01, *p < 0.05.

In contrast, the CRD group demonstrated more limited improvements, with significant changes only in BG levels at 30 min and 2 h (*p* = 0.017). There were no significant changes in insulin levels, C-peptide levels, insulin sensitivity, or lipid levels. Despite the significant improvements observed in multiple metabolic parameters within the PB-CRD group, the between-group comparisons of the changes in glucose and lipid metabolism did not reach statistical significance (*p* > 0.05) (Supplementary Table S6; Figure 3).

### Liver and kidney function

**Figure 4:**
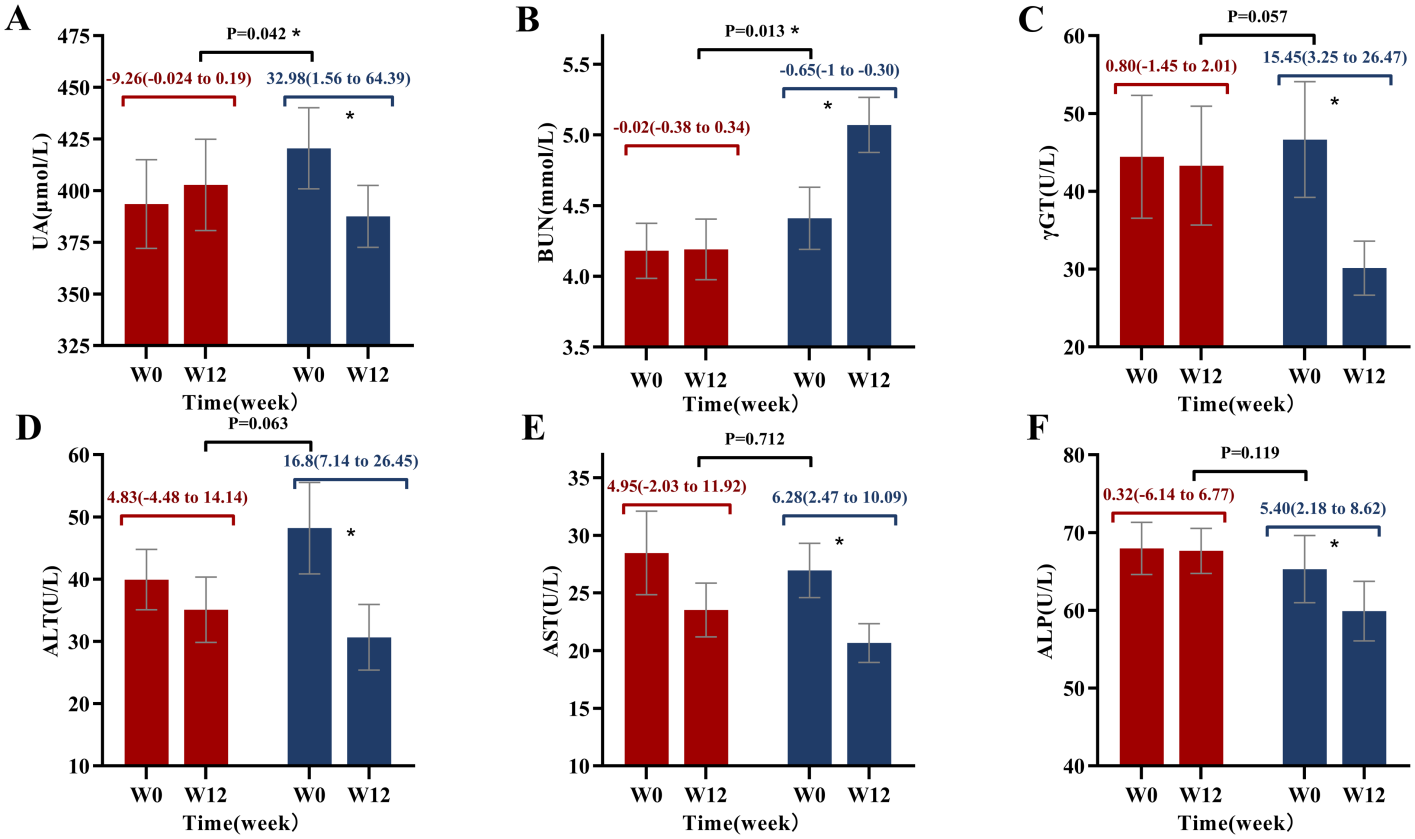
Changes in liver and kidney function from baseline to week 12. **(A)** Uric acid (UA); **(B)** blood urea nitrogen (BUN); **(C)** gamma-glutamyl transferase (*γ*-GT); **(D)** alanine aminotransferase (ALT); **(E)** aspartate aminotransferase (AST); **(F)** alkaline phosphatase (ALP); *p < 0.05.

Following 12 weeks of intervention, the PB-CRD group exhibited significant reductions in UA levels (32.98 [95% CI: 1.56 to 64.39], *p* < 0.05) and an increase in BUN (0.65 [95% CI: 0.30 to 1.00], *p* < 0.05). These changes showed significant intergroup differences when compared to the CRD group. Furthermore, liver function markers in the PB-CRD group, including ALT, AST, GGT, and ALP, demonstrated significant reductions (all *p* < 0.05), suggesting improved liver health following the intervention. However, when comparing the PB-CRD group to the CRD group, no significant differences were observed in these parameters (Supplementary Table S6; Figure 4).

### Oxidative stress and inflammation

**Figure 5:**
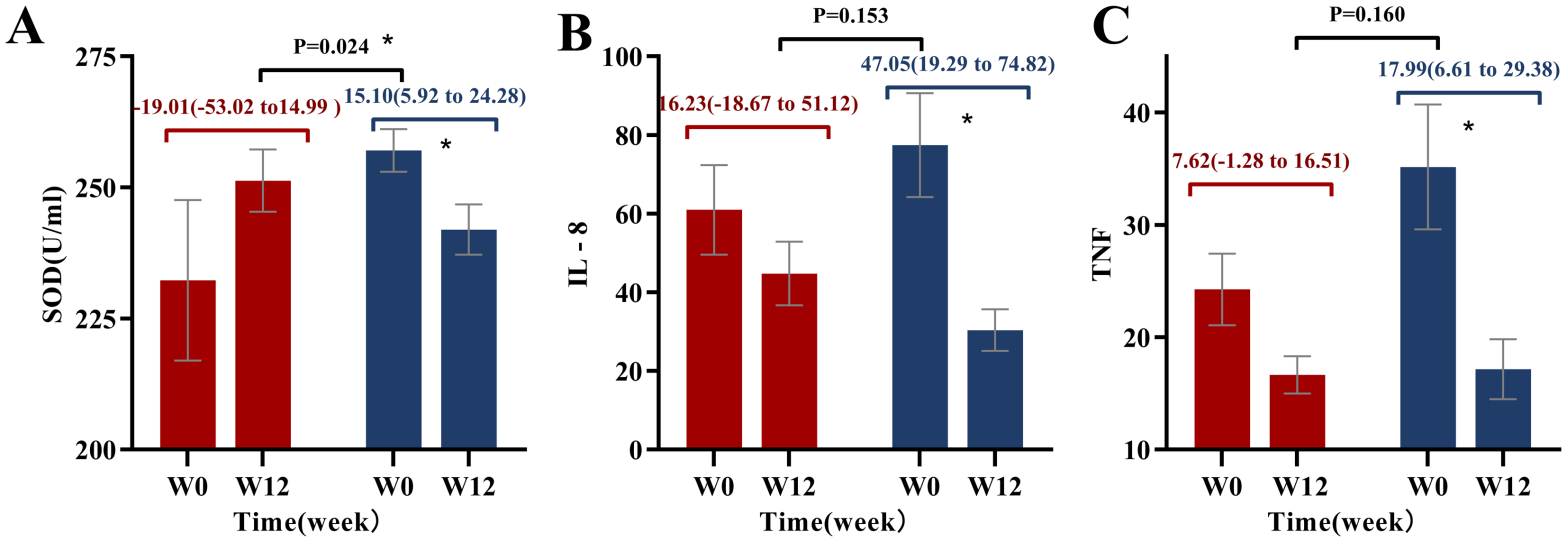
Changes in antioxidant and inflammatory markers from baseline to week 12. **(A)** Superoxide dismutase (SOD); **(B)** interleukin-8 (IL-8); **(C)** tumor necrosis factor (TNF); *p < 0.05.

As shown in Figure 5 and Supplementary Table S6, after 12 weeks of intervention, SOD levels significantly decreased in the PB-CRD group (15.10 [95% CI: 5.92 to 24.28], *p* < 0.05), indicating a greater change compared to the CRD group, highlighting PB-CRD’s stronger impact on oxidative stress. In addition, TNF-*α* and IL-8 levels significantly decreased in the PB-CRD group (*p* < 0.05), indicating the diet’s anti-inflammatory effects. However, no significant differences were observed between the two groups for these markers.

### Adverse events

No deaths or serious adverse events were reported in this study. Additionally, participants did not experience any mild adverse reactions such as fatigue, dizziness, headache, upper abdominal pain, dyspepsia, or constipation.

## Discussion

CRDs are widely used for weight management; however, different CRD approaches generally produce similar, limited outcomes. Studies indicate that time-restricted eating does not outperform daily calorie restriction in reducing body weight or improving metabolic risk factors (25). Despite equivalent calorie restriction, varying dietary patterns may produce different results due to factors like food composition and individual differences. For instance, low-carbohydrate diets have been shown to improve lipid profiles in patients that are overweight or obesity (26), while Mediterranean diets enhance insulin sensitivity and glucose regulation, particularly in adults with prediabetes or metabolic syndrome (22). In this 12-week intervention, the PB-CRD group lost an average of 6.56 kg, compared to 5.11 kg in the traditional CRD group, with a significantly greater reduction in body fat percentage (2.96% vs. 0.81%, *p* = 0.028). Previous studies found that participants following daily caloric restriction lost an average of 5.1 kg over 12 weeks (27), while those adhering to a vegan diet experienced a 7.5% weight loss over 6 months (20). Unlike conventional approaches, our study demonstrates that the PB-CRD functions through a dual-action mechanism: the synergy between caloric deficit and structural dietary optimization. This combined strategy likely accounts for the numerically superior weight loss and the significantly greater reduction in body fat percentage compared to traditional caloric restriction.

This greater reduction in body fat percentage provided a physiological foundation for the metabolic improvements observed in the PB-CRD group. The decrease in TC levels aligns with previous studies, indicating that vegetarians typically have lower TC levels (28, 29). Additionally, improvements in insulin sensitivity indicators (such as HOMA-IR, Matsuda index, AUCins/AUCBG, and C-peptide) may be associated with the fiber content in PBDs. Previous research suggests that dietary fiber helps delay postprandial blood sugar increases, reduces insulin demand, and lowers the risk of T2D (30). These metabolic improvements were accompanied by a decrease in UA levels, potentially reflecting reduced UA production due to enhanced insulin sensitivity and reduced fat accumulation (31, 32). Simultaneously, the increase in BUN levels suggests an increased metabolic processing of plant-based proteins, consistent with the dietary shift (33).

The significant rise in SOD levels in the PB-CRD group indicates improved antioxidant capacity, which likely contributed to the alleviation of oxidative stress (20, 34). Correspondingly, this enhanced antioxidant status modulated inflammatory signaling, where the notable reduction in inflammatory markers (TNF-*α* and IL-8) further suggests a role in inhibiting the production of pro-inflammatory cytokines and improving chronic low-grade inflammation (35). Consequently, the reduction in oxidative stress and inflammation likely contributed to the decreased liver enzymes (such as ALT, AST, GGT, ALP), thereby suggesting a positive impact of the intervention on hepatic function (36, 37).

While both groups were subjected to caloric restriction, the superior metabolic improvements observed in the PB-CRD group—despite equivalent caloric targets—highlight the specific therapeutic value of plant-based dietary components. PBDs provide benefits through several physiological mechanisms. First, the high fiber content in PBDs, characterized by low energy density and high nutrient density, facilitates the consumption of larger food volumes, regulates calorie intake, increases satiety, and promotes weight loss (38, 39). It also decelerates gastric emptying, stabilizes BG levels, and reduces insulin demand, thereby lowering the risk of T2D (40, 41). Second, PBDs exhibit a higher thermic effect of food, which promotes protein synthesis, increases energy expenditure, and elevates basal metabolic rate, consequently enhancing fat oxidation and supporting weight loss (42, 43). Third, PBDs modulate gut microbiota, particularly by increasing *Faecalibacterium prausnitzii*, which promotes the production of short-chain fatty acids (SCFAs). These SCFAs, especially propionate, butyrate, and acetate, improve insulin sensitivity, exert anti-inflammatory effects (44–46), and also regulate appetite and energy balance through the activation of GPR41 and GPR43 receptors, consequently stimulating GLP-1 secretion (47). (4) PBDs are rich in polyphenolic compounds, such as flavonoids and phenolic acids, which act as antioxidants, reducing oxidative stress and inflammation by inhibiting NF-κB and NLRP3 inflammasome pathways (48).

This study has some limitations. First, the 12-week duration may be insufficient to evaluate the long-term sustainability of the observed benefits. Second, the single-center design and small sample size limit the generalizability of the findings and reduce the statistical power, potentially affecting the detection of significant differences. Third, reliance on self-reported dietary intake for calorie calculation introduces the risk of reporting bias, which may affect the accuracy of the data. Fourth, the differing management protocols may have caused unequal dietary standardization and intervention intensity between groups. Lastly, we acknowledge the susceptibility of the non-inferiority design to the ‘dilution effect.’ In this study, high dropout rates exacerbated by the COVID-19 pandemic led to a dilution effect of 0.62 kg, as evidenced by discrepancies between PPS and ITT analyses. Although dropout rates were evenly distributed between groups, they likely masked the true efficacy of the dietary intervention, highlighting that clinical effectiveness remains heavily contingent upon long-term dietary adherence. These findings point to the need for future large-scale, multi-center trials with extended follow-up and rigorous monitoring to enhance the robustness and generalizability of the results.

In conclusion, this 12-week study demonstrated that the PB-CRD achieved weight loss comparable to the traditional CRD, with non-inferiority confirmed. Furthermore, the PB-CRD group showed notable improvements in body composition, insulin sensitivity, liver function, antioxidant capacity, and inflammatory markers. These findings suggest that PB-CRD is non-inferior to traditional CRD in weight loss efficacy and may offer potential metabolic health benefits. This highlights its potential as a viable dietary approach for obesity management and metabolic disease prevention.

## Data Availability

The original contributions presented in the study are included in the article/Supplementary material, further inquiries can be directed to the corresponding authors.
