# Supplementary material for: Plant-based caloric restriction diets versus conventional calorie-restricted diets for weight loss and metabolic health in obese adults: a 12-week randomized, open-label, non-inferiority trial
Source: Front Nutr. 2026 Apr 13;13:1805225. doi: 10.3389/fnut.2026.1805225 (PMC13111110; doi:10.3389/fnut.2026.1805225)
Supplement: Supplementary file 1 [file Data_Sheet_1.zip › Supplementary Table 6.DOCX]

**Table S6.** Comparison of secondary outcomes between the PB-CRD and CRD groups at 12 weeks

| **Variable** | **PB-CRD Group Change**  **(Baseline - Week 12)** | **CRD Group Change**  **(Baseline - Week 12)** | **Between-group difference** | ***P* value**^b^ |
| --- | --- | --- | --- | --- |
| hbA1c (%) | 0.06(-0.04-0.17) | 0.09(-0.01-0.18) | -0.03 (-0.17 - 0.11) | 0.720 |
| FBG (mmol/l) | 0.04(-0.23-0.31) | 0.01(-0.2-0.23) | 0.03 (-0.32 - 0.38) | 0.882 |
| 0.5hBG (mmol/l) | 0.84(0.34-1.34) | 0.66(-0.02-1.34) | 0.18 (-0.68 – 1.04) | 0.649 |
| 1hBG (mmol/l) | 0.55(-0.06-1.15) | 0.24(-0.54-1.03) | 0.31 (-0.65 - 1.27) | 0.520 |
| 2hBG (mmol/l) | 0.46(-0.29-1.2) | 0.24(-0.82-1.29) | 0.22 (-1.01 – 1.45) | 0.719 |
| 3hBG (mmol/l) | 0.30(-0.17-0.77) | -0.68(-1.2-0.16) | 0.98 (0.31 - 1.65) | 0.006* |
| FINS (mU/L) | -4.92(-21.45-11.6) | 2.06(-4.94-0.83) | -6.98 (-24.71 - 10.75) | 0.431 |
| 0.5hINS (mU/L) | 7.01(-11.13-25.16) | 28.08(-6.16-62.31) | -21.07 (-57.04 - 14.90) | 0.242 |
| 1hINS (mU/L) | 16.01(-12.78-44.8) | -13.71(-51.32-23.89) | 29.72 (-15.68 - 75.12) | 0.192 |
| 2hINS (mU/L) | 22.19(-1.13-45.5) | 24.96(-22.65-72.57) | -2.77 (-50.81 - 45.27) | 0.909 |
| 3hINS（mU/L） | 6.69(-5.84-19.21) | -6.3(-19.53-6.93) | 12.99 (-4.81 – 30.79) | 0.146 |
| FCP (ng/ml) | 0.22(-0.56-1.00) | -0.04(-0.39-0.30) | 0.26 (-0.64 – 1.16) | 0.554 |
| 0.5hCP (ng/ml) | 5.22(-3.44-13.87) | 1.24(-0.36-2.84) | 3.98 (-5.28 – 13.24) | 0.397 |
| 1hCP (ng/ml) | 2.99(1.23-4.75) | -0.29(-1.85-1.27) | 3.28 (0.06 – 6.50) | 0.046* |
| 2hCP (ng/ml) | 1.67(0.64-2.70) | 0.52(-1.23-2.28) | 1.15 (-0.72 – 3.02) | 0.228 |
| 3hCP（ng/ml） | 1.03(0.27-1.79) | -0.79(-2.01-0.44) | 1.82(0.45–3.19) | 0.010* |
| HOMA-IR | 0.81(0.21–1.42) | -0.10(-1.35–1.15) | 0.91(-0.56–2.38) | 0.292 |
| Matsuda Index | -20.11(-34.34–-5.87) | -0.63(-10.45–9.18) | -19.48(-36.19–-2.77) | 0.024 |
| AUCins/AUCBG | 0.09(0.07–0.18) | 0.07(-0.09–0.22) | 0.02(-0.16–0.20) | 0.756 |
| HOMA-β | 6.81(-6.49–20.11) | -3.03(-20.16–14.05) | 9.84(-11.13–30.81) | 0.347 |
| UA (umol/L) | 32.98(1.56-64.39) | -9.26(-35.11-16.58) | 42.24(1.57–82.91) | 0.042* |
| BUN (mmol/l) | 2.04(-0.74-4.82) | -1.01(-3.46-1.43) | 3.05 (-0.64 – 6.74) | 0.105 |
| TC (mmol/L) | 0.25(0.01-0.48) | -0.03(-0.24-0.19) | 0.28 (-0.01 – 0.57) | 0.055 |
| TG (mmol/L) | 0.18(-0.13-0.49) | 0.23(-0.03-0.49) | -0.05 (-0.45 – 0.35) | 0.797 |
| HDL (mmol/L) | 0(-0.06-0.06) | 0.02(-0.03-0.07) | -0.02 (-0.10 – 0.06) | 0.658 |
| LDL (mmol/L) | 0.16(-0.07-0.39) | -0.12(-0.29-0.06) | 0.28 (-0.02 – 0.58) | 0.066 |
| ALT (U/L) | 16.8(7.14-26.45) | 4.83(-4.48-14.14) | 11.97 (-0.65 – 24.59) | 0.063 |
| AST (U/L) | 6.28(2.47-10.09) | 4.95(-2.03-11.92) | 1.33 (-7.06 – 9.72) | 0.712 |
| GGT (U/L) | 15.45(3.25-26.47) | 0.80(-1.45-2.01) | 14.65 (-0.46 – 29.76) | 0.057 |
| ALP (U/L) | 5.40(2.18-8.62) | 0.32(-6.14-6.77) | 5.08 (-1.33 – 11.49) | 0.119 |
| SOD (U/ml) | 15.10(5.92-24.28) | -19.01(-53.02-14.99) | 34.11 (4.65 – 63.57) | 0.024* |
| IL-6 | 0.30(-1.12-1.72) | 0.05 (-1.31-1.41) | 0.25 (-1.71 – 2.21) | 0.800 |
| IL-8 | 47.05(19.29-74.82) | 16.23(-18.67-51.12) | 30.82 (-11.75 – 73.39) | 0.153 |
| TNF | 17.99(6.61-29.38) | 7.62(-1.28-16.51) | 10.37 (-4.18 – 24.92) 24.92) | 0.160 |
| hbA1c, hemoglobin A1c; FBG, fasting blood glucose; 0.5hBG, 0.5-hour blood glucose; 1hBG, 1-hour blood glucose; 2hBG, 2-hour blood glucose; 3hBG, 3-hour blood glucose; FINS, fasting insulin; 0.5hINS, 0.5-hour insulin; 1hINS, 1-hour insulin; 2hINS, 2-hour insulin; 3hINS, 3-hour insulin; FCP, fasting C-peptide; 0.5hCP, 0.5-hour C-peptide; 1hCP, 1-hour C-peptide; 2hCP, 2-hour C-peptide; 3hCP, 3-hour C-peptide; HOMA-IR, homeostasis model assessment of insulin resistance; Matsuda Index, Matsuda insulin sensitivity index; AUCins/AUCBG, ratio of area under the curve for insulin to area under the curve for glucose; HOMA-β, homeostasis model assessment of beta cell function; UA, uric acid; BUN, blood urea nitrogen; TC, total cholesterol; TG, triglycerides; HDL, high-density lipoprotein cholesterol; LDL, low-density lipoprotein cholesterol; ALT, alanine aminotransferase; AST, aspartate aminotransferase; GGT, gamma-glutamyl transferase; ALP, alkaline phosphatase; SOD, superoxide dismutase; IL-6, interleukin-6; IL-8, interleukin-8; TNF, tumor necrosis factor.  Between-group difference = (PB-CRD change) – (CRD change), assessed by repeated measures ANOVA  Changes from baseline to Week 12 and between-group differences are expressed with 95% confidence intervals (CI).  P-values evaluate the statistical significance of differences between groups. * p < 0.05. | | | | |
