# Supplementary material for: Plant-based caloric restriction diets versus conventional calorie-restricted diets for weight loss and metabolic health in obese adults: a 12-week randomized, open-label, non-inferiority trial
Source: Front Nutr. 2026 Apr 13;13:1805225. doi: 10.3389/fnut.2026.1805225 (PMC13111110; doi:10.3389/fnut.2026.1805225)
Supplement: Supplementary file 1 [file Data_Sheet_1.zip › Supplementary Table 1.docx]

**Table S1** Reasons for Drop-out in PB-CRD and CRD Groups

| **Group** | **Category** | **Specific Reasons** | **N** |
| --- | --- | --- | --- |
| **PB-CRD** | Voluntary Withdrawal | - Relocation to another city | 1 |
|  |  | - Returning to hometown | 1 |
|  |  | - Fatigue and poor health after COVID-19 | 1 |
|  | Non-compliance  (adherence rate below 50%) | - Unable to adapt to the dietary intervention | 1 |
|  |  | - Failure to complete required records | 3 |
|  | Pregnancy | - Pregnant during the intervention | 1 |
|  | Lost Contact | - Unable to reach participants (likely related to relocation, work obligations, or lack of communication) | 2 |
|  | Other Reasons | - Family issues (e.g., a sick family member) | 1 |
|  |  | - Fracture | 1 |
|  |  | **Total Drop-outs** | **12** |
| **CRD** | Voluntary Withdrawal | - Personal reasons | 2 |
|  |  | - Post-COVID myocarditis | 1 |
|  | Non-compliance  (adherence rate below 50%) | - Failure to complete required records | 2 |
|  |  | - Difficulty adhering to dietary plan due to lingering post-COVID symptoms | 2 |
|  |  | - Fatigue and poor health after COVID-19 | 1 |
|  | Lost Contact | - Unable to reach participants (likely related to relocation, work obligations, or lack of communication) | 1 |
|  | No Evaluated Data | No available data due to early withdrawal | 6 |
|  |  | **Total Drop-outs** | 16 |

### Drop-out Rate Comparison

PB-CRD: 12/40 = 30.00%

CRD: 16/40 = 40.00%

Statistical comparison of drop-out rates (using chi-square or Fisher's exact test): P = 0.348
