# Supplementary material for: Plant-based caloric restriction diets versus conventional calorie-restricted diets for weight loss and metabolic health in obese adults: a 12-week randomized, open-label, non-inferiority trial
Source: Front Nutr. 2026 Apr 13;13:1805225. doi: 10.3389/fnut.2026.1805225 (PMC13111110; doi:10.3389/fnut.2026.1805225)
Supplement: Supplementary file 1 [file Data_Sheet_1.zip › Supplementary Table 2.docx]

**Table S2** Dropout Population Participation Time Distribution

| Time Interval | PB-CRD | CRD |
| --- | --- | --- |
| 0 (Not Evaluable) | 0 | 6 |
| <3 weeks | 4 | 6 |
| 3 weeks - <6 weeks | 5 | 2 |
| ≥6 weeks | 3 | 2 |
