# Supplementary material for: Plant-based caloric restriction diets versus conventional calorie-restricted diets for weight loss and metabolic health in obese adults: a 12-week randomized, open-label, non-inferiority trial
Source: Front Nutr. 2026 Apr 13;13:1805225. doi: 10.3389/fnut.2026.1805225 (PMC13111110; doi:10.3389/fnut.2026.1805225)
Supplement: Supplementary file 1 [file Data_Sheet_1.zip › Supplementary Table 3, 4, and 5.docx]

**Table S3** Correlation Analysis Between Weight Change and Days of Participation

| Variable | Pearson Correlation Coefficient (r) | p-value | Sample Size (N) |
| --- | --- | --- | --- |
| Weight Change & Participation Days | 0.471* | 0.027 | 22 |

**Table S4** Dilution Effect Between PPS and ITT Results

| Analysis Type | PB-CRD Weight Change (kg) | CRD Weight Change (kg) | Between-Group Difference (kg) | Dilution Effect (kg) | Relative Reduction (%) |
| --- | --- | --- | --- | --- | --- |
| PPS | -6.56 | -5.11 | 1.44 | N/A | N/A |
| ITT | -5.34 | -4.51 | 0.82 | 0.62 | 0.62 |

**Table S5** Comparison of Weight Change Between Dropouts and Completers

| Intervention Group | Status | Mean (kg) | SD (kg) | p (two-tailed) | Mean Difference (95% CI) |
| --- | --- | --- | --- | --- | --- |
| PBCRD | Completers | 6.47 | 2.90 | < 0.001 | 3.75 (1.91, 5.59) |
|  | Dropouts | 2.72 | 1.81 |  |  |
| CRD | Completers | 5.17 | 2.59 | 0.017 | 2.22 (0.43, 4.02) |
|  | Dropouts | 2.95 | 1.50 |  |  |
