# Supplementary figures and images for: Plant-based caloric restriction diets versus conventional calorie-restricted diets for weight loss and metabolic health in obese adults: a 12-week randomized, open-label, non-inferiority trial
Source: Front Nutr. 2026 Apr 13;13:1805225. doi: 10.3389/fnut.2026.1805225 (PMC13111110; doi:10.3389/fnut.2026.1805225)

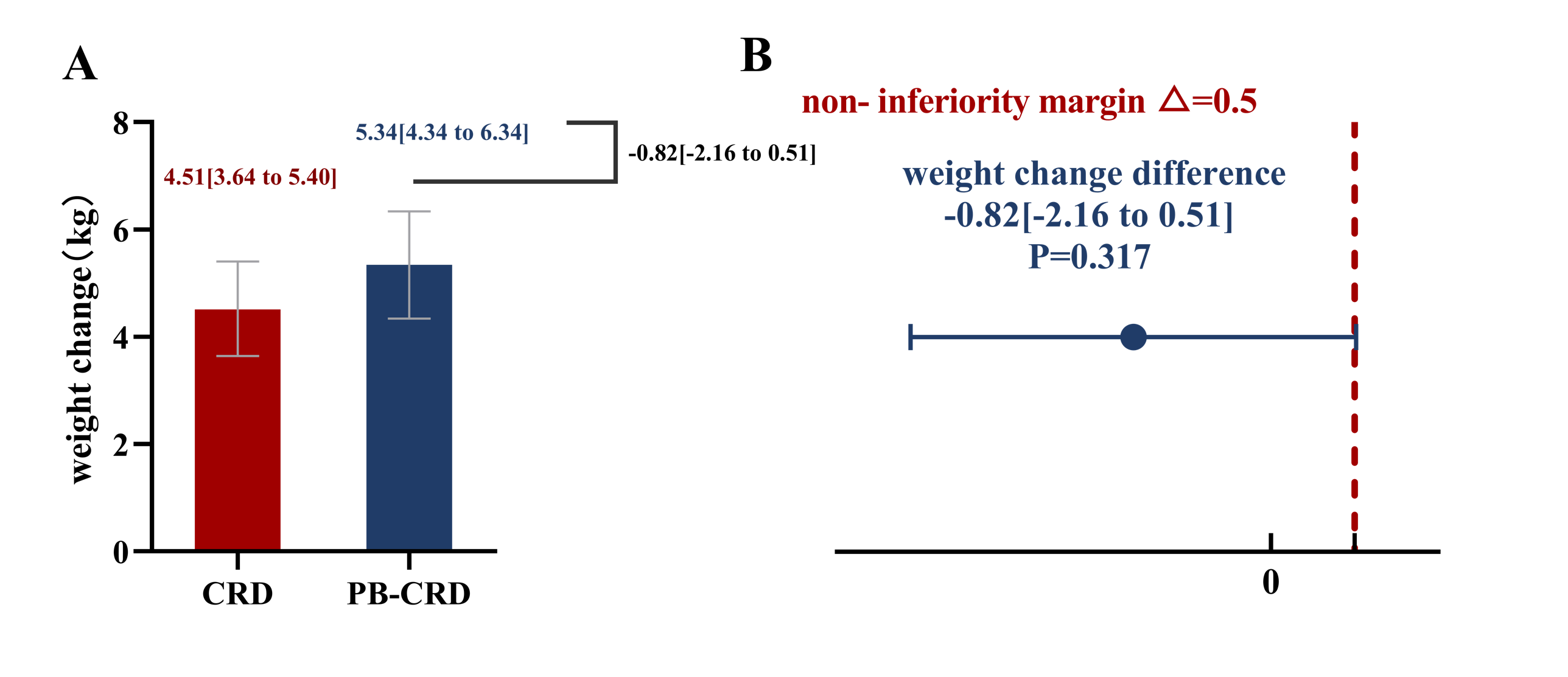

Supplement: Supplementary file 2 [file Image_1.tif]

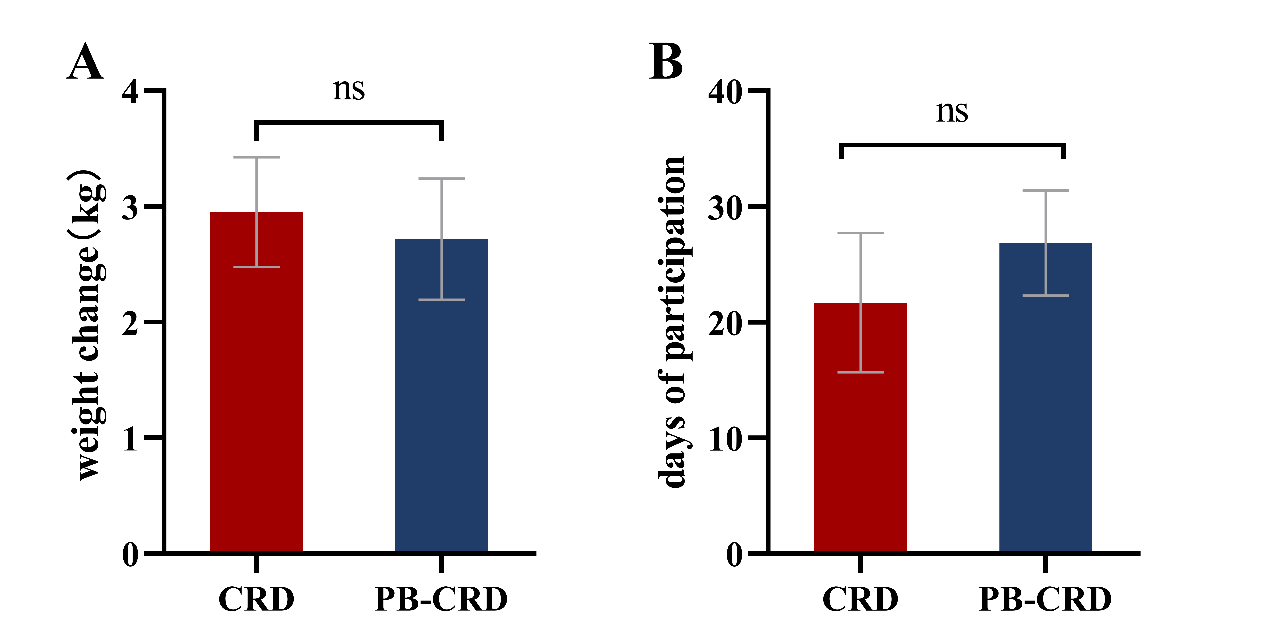

Supplement: Supplementary file 3 [file Image_2.png]
