# Supplementary material for: Plant-based caloric restriction diets versus conventional calorie-restricted diets for weight loss and metabolic health in obese adults: a 12-week randomized, open-label, non-inferiority trial
Source: Front Nutr. 2026 Apr 13;13:1805225. doi: 10.3389/fnut.2026.1805225 (PMC13111110; doi:10.3389/fnut.2026.1805225)
Supplement: Supplementary file 4 [file Supplementary_file_1.docx]

**Supplementary Material 1 Variable Calculation Formulas**

1. **The Area Under the Curve (AUC) Calculation for Plasma Glucose, Insulin, and C-peptide**

The AUC for plasma glucose (AUCBG), insulin (AUCI), and C-peptide (AUCC) was calculated using the trapezoidal method:

**AUC for plasma glucose (AUCBG):**

AUC-BG=0.5×[BG_0​_+BG_30_​]+0.5×[BG_30_​+BG_60_​]+1×[BG_60_​+BG_120​_]+0.5×[BG_120_​+BG_180​_]

where BG_time_​ represents blood glucose concentration at the specified time points (in mmol/L).

**AUC for insulin (AUC-I):**

AUCins=0.5×[Ins_0_​+Ins_30​_]+0.5×[Ins_30_​+Ins_60_​]+1×[Ins_60_​+Ins_120_]+0.5×[Ins_120​_+Ins_180_]

where Ins_time_  represents insulin concentration at the specified time points (in mU/L)

**AUC for C-peptide (AUCC):**

AUCC=0.5×(C0+2×C30​)+0.5×(C30​+3×C60​)+(C60​+4×C120​)+0.5×(C120​+2×C180​)

where Ctime​ represents C-peptide concentration at the specified time points (in ng/ml).

1. **Homeostatic Model Assessment of Insulin Resistance (HOMA-IR)**

HOMA-IR was calculated from the fasting measurements using the following formula^1^:

HOMA-IR=(Ins0*BG0)/405

Where

Ins0​ represents fasting insulin concentration (in mU/L).

BG0​ represents fasting blood glucose concentration (in mmol/L).

1. **Matsuda Index of Insulin Sensitivity**

The Matsuda index was calculated using the formula previously published ^2^:

Matsuda Index =10,000/√ (BG0 × Ins0 × mean BG × mean Ins )

Where:

BG0​ is the fasting blood glucose concentration (in mmol/L).

Ins0​ is the fasting insulin concentration (in mU/L).

mean BG is the average blood glucose during the OGTT (in mmol/L).

mean Ins is the average insulin during the OGTT (in mU/L).

1. **AUC Insulin/Glucose ratio**

The AUC Insulin/Glucose ratio is used to evaluate the relationship between insulin secretion and blood glucose levels. It is calculated as:

AUC-Ins/AUC-BG=AUCIns/AUCBG

Where:

AUC-Ins represents the area under the insulin concentration curve.

AUC-BG represents the area under the glucose concentration curve.

1. **Homeostasis Model Assessment of Beta-Cell Function(HOMA-β）**

HOMA-β is used to estimate pancreatic beta-cell function based on fasting insulin and glucose levels. It is calculated as ^3^:

HOMA-β=(360×Ins0)/(BG0​−63)​

Where:

Ins0 represents fasting insulin concentration (in mU/L).

BG0 represents fasting blood glucose concentration (in mmol/L).

**Reference：**

1. Matthews DR, Hosker JP, Rudenski AS, Naylor BA, Treacher DF, Turner RC. Homeostasis model assessment: insulin resistance and beta-cell function from fasting plasma glucose and insulin concentrations in man. *Diabetologia*. Jul 1985;28(7):412-9. doi:10.1007/bf00280883

2. Matsuda M, DeFronzo RA. Insulin sensitivity indices obtained from oral glucose tolerance testing: comparison with the euglycemic insulin clamp. *Diabetes Care*. Sep 1999;22(9):1462-70. doi:10.2337/diacare.22.9.1462

3. Sosenko JM, Skyler JS, DiMeglio LA, et al. A new approach for diagnosing type 1 diabetes in autoantibody-positive individuals based on prediction and natural history. *Diabetes Care*. Feb 2015;38(2):271-6. doi:10.2337/dc14-1813
